# Child and Adolescent Mental Health Training Programs for Non-specialist Mental Health Professionals in Low and Middle Income Countries: A Scoping Review of Literature

**DOI:** 10.1007/s10597-021-00805-w

**Published:** 2021-03-02

**Authors:** Vijay Raj, Vibhay Raykar, Ainsley M. Robinson, Md Rafiqul Islam

**Affiliations:** 1 Goulburn Valley Health, Graham Street, Shepparton, VIC 3630 Australia; 2 Department of Rural Health, The University of Melbourne, Shepparton, VIC 3630 Australia; 3 Rural Health School, La Trobe University, Shepparton, VIC 3630 Australia; 4 Goulburn Valley Area Mental Health Services, Goulburn Valley Health, Graham Street, Shepparton, VIC 3630 Australia

**Keywords:** Child and adolescent mental health (CAMH), Training, LMICs, Non-specialist

## Abstract

Large treatment deficits in child and adolescent mental health (CAMH) care exist in low and middle income countries (LMICs). This study reviewed CAMH training programs for non-specialist health professionals (NSHPs) in LMICs. Multiple databases were searched for peer-reviewed articles describing programs from 2005 to 2018. Educational source materials, trainee evaluation methods, and perspectives on teaching methods, course content and scheduling were studied. Six programs were identified. NSHPs were most appreciative of training which included case-based discussions, role plays and clinical demonstrations that were relevant to local contexts. A need for less intense and more flexible timetables to enable reflection was identified. WHO’s mental health gap action program intervention guide (mhGAP-IG) and international association of child and adolescent psychiatrists and allied professionals resources should be used; they are free, easily accessible, and developed with extensive international contributions. Additionally, mhGAP-IG assessment tool encourages mutual learning, thereby iteratively enhancing training programs.

## Introduction

Child and adolescent mental health (CAMH) is a major concern worldwide due to the high prevalence of psychiatric disorders in this group; globally, up to 20% of children and adolescents suffer from a mental illness and up to half of adult mental health disorders originate in childhood (Klasen & Crombag, 2013; Morris, 2011; WHO, 2005). The mental health of children and adolescents in low and middle income countries (LMICs) is of particular concern for several reasons. Firstly, the majority of the world’s children and adolescents (90%) live in LMICs, where they typically constitute about half of the total population in these countries (Barry et al., 2013; Clark, 2020; Patel et al., 2008). Secondly, there is a severe lack of trained mental health professionals in LMICs, including specific CAMH trained professionals (Juengsiragulwit, 2015; Morris et al., 2011; WHO, 2005). Thirdly, children and adolescents living in these countries are more likely to be exposed to environmental risk factors such as trauma, substance misuse, poverty, and inadequately treated physical and mental disabilities that increase their vulnerability to mental health disorders (Belfer, 2008; Pedersen, 2019).

The World Health Organisation’s (WHO) child mental health atlas, published in 2005, was conceived in a collaborative effort between the WHO, the world psychiatric association’s presidential global programme on child mental health and the international association for child and adolescent psychiatry and allied professions (IACAPAP) with the aim of highlighting the deficits in CAMH resources globally and to encourage further development of CAMH policy, services and training (WHO, 2005). The report stated that half to two thirds of all CAMH needs remained unmet in most countries; the treatment gap between those who need mental health interventions and those who receive such healthcare is considerable (WHO, 2005). This gap is significantly larger in LMICs. Furthermore, a significant deficiency in training initiatives for primary care providers in CAMH disorders compared to adult mental health disorders in LMICs has been highlighted (WHO, 2005). A major barrier to closing the treatment gap in LMICs is the meagre availability of skilled CAMH human resources, as well as unequal and inefficient dissemination and application of resources which are accessible (Babatunde et al., 2019; Saxena et al., 2007).

Several studies have proposed solutions for closing the treatment gap which lie in the adequate training and task shifting of mental health interventions to non-specialist health professionals (Bruckner, 2011; Juengsiragulwit, 2015; Murray et al., 2011; O’Brien et al., 2016; Servili, 2012; Woods-Jaeger et al., 2017). This involves the shifting of some of the tasks performed typically by specialist mental health professionals to non-specialist health workers with the hope of increasing accessibility for children and adolescents to timely and appropriate mental health assessment and care. These include nurses, general practitioners, general healthcare workers, and other medical professionals involved in rural and regional outreach. To successfully carry out task shifting and to ensure high quality care, adequate training is essential (Agyapong et al., 2016; Ola & Atilola, 2019; Shahmalak et al., 2019). Furthermore, to strengthen task shifting in resource-limited settings, it is preferrable that training programs are developed by CAMH professionals (Galagali & Brooks, 2020). This study aims to review these efforts with the goal of understanding some of the strengths and limitations of previous CAMH training programs in LMICs. This may be invaluable in the appropriate development of CAMH training initiatives in LMICs for general health professionals in the future.

## Objectives

### General Objective

To identify peer-reviewed articles published between 2005 and 2018, and qualitatively evaluate key elements of CAMH training courses for non-specialist health professionals in LMICs.

### Specific Objectives

To understand and critically evaluate:

- (a) the educational source materials used,
- (b) the perspectives on teaching methods,
- (c) the perspectives on course schedule,
- (d) the course content, and
- (e) the tools used for trainee evaluation.

## Methods

A scoping study methodology was undertaken to identify and qualitatively review current research findings (Arksey & O’Malley, 2005). This study focused on searching peer-reviewed research reports. CINAHL, PsycINFO, Cochrane, Embase and Medline databases were searched for original research articles, scientific reviews or reports that were published between 2005 (the year the WHO child mental health atlas was published) and 2018, in the English language. The ‘Search Terms’ that were used to identify relevant articles were:

- (child OR adolesc* OR youth OR paediatr*) AND (training OR education OR upskilling OR workshop OR in-service*) AND (lamic OR lmic OR lami OR “developing countries” OR “low and middle income countries” OR “low income countries”) and (“mental health” OR psychiatr* OR icamh OR mhgap OR mhgap-ig OR iacapap OR camh* OR cymh*)
- Manual searching of articles from the references and citations was also conducted. The papers were selected on the basis of the following eligibility criteria and the search limits to both abstracts and full text articles.

### Inclusion Criteria

- (a) Published studies.
- (b) Training Programs in General CAMH in LMICs.
- (c) Training for non-specialist mental health professionals.
- (d) Information on type of training program including course content.
- (e) Information on duration of training program.
- (f) Information on teaching methods used.
- (g) Information on trainee and/or course evaluation.

### Exclusion Criteria

- (a) Unpublished information/working papers/reports.
- (b) CAMH training programs in Non-LMICs.
- (c) CAMH training only for specialist mental health workers.
- (d) No information on type of training program including course content.
- (e) No information on duration of program.
- (f) No information on teaching methods used.
- (g) No information on course or trainee evaluation.

Additionally, non-peer-reviewed papers, for example, conference or meeting proceedings, working papers or reports, monographs, book chapters, theses and dissertations were excluded.

## Results

**Fig. 1:**
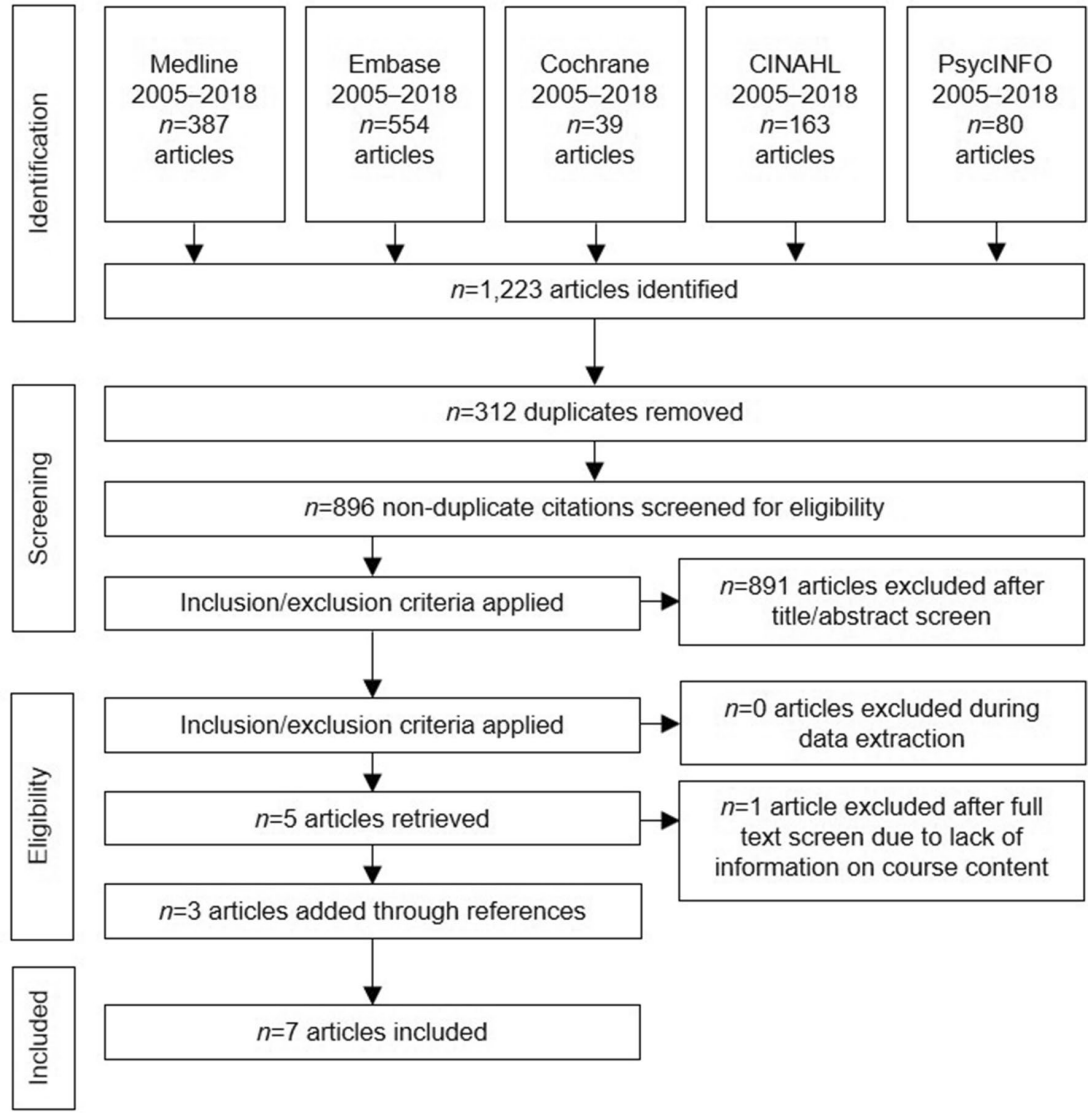
Flowchart of literature search

The initial search identified 1223 references, of which 312 duplicates were removed. Eligibility criteria were applied to the remaining 896 papers. A further 892 papers that did not meet our criteria were excluded, but three more were included through manual searching of references. Seven studies were included in the final review. Figure 1 shows the flow diagram of the search. Table 1 shows that the studies included described programs in the LMICs: Brazil (Blanco-Vieira et al., 2017), Uganda (Akol et al., 2017), Nigeria (Omigbodun et al., 2007), India (Dogra et al., 2005; Russell & Nair, 2010a, 2010b), and Ethiopia (Tesfaye et al., 2014). Of these seven articles included, three of them were focused in India, of which two described the same program, therefore in total, there were six different programs from five LMICs that were included in this study.

**Table 1:** Included studies

Study title	Year	LMIC	Name of course	Program designation
An evaluation of a collaborative course for child and adolescent mental health professionals (Blanco-Vieira et al., 2017)	2017	Brazil	Child and adolescent mental health specialization course (CESMIA)	The Brazilian program
Does child and adolescent mental health in-service training result in equivalent knowledge gain among cadres of non-specialist health workers in Uganda? A pre-test post-test study (Akol et al., 2017)	2017	Uganda	WHO’s mental health gap action programme (mhGAP) intervention guide (IG) + PowerPoint presentations from the IACAPAP textbook	The Ugandan program
Training health professionals for child and adolescent mental health care in Nigeria: a qualitative analysis (Omigbodun et al., 2007)	2007	Nigeria	Course in child and adolescent mental health by The West African College of physicians (Nigeria Chapter), faculty of psychiatry	The Nigerian program
Training CAMHS professionals in developing countries: an Indian case study (Dogra et al., 2005)	2005	India	Modified course on child mental health based on child psychiatry training for medical undergraduates at University of Leicester, UK	The Varanasi Indian program
Strengthening the paediatricians project 1: the need, content and process of a workshop to address the priority mental health disorders of adolescence in countries with low human resource for health (Russell & Nair, 2010a)	2010	India	Strengthening the paediatricians project (SAPP) as part of the postgraduate diploma in adolescent paediatrics by child development centre in the University of Kerala, India	The SAPP Indian program
Strengthening the paediatricians project 2: the effectiveness of a workshop to address the priority mental health disorders of adolescents in low-health related human resource countries (Russell & Nair, 2010b)	2010	India	SAPP as part of the postgraduate diploma in adolescent paediatrics by child development centre in the University of Kerala, India	The SAPP Indian program
The development of a model of training in child psychiatry for non-physician clinicians in Ethiopia (Tesfaye et al., 2014)	2014	Ethiopia	Child and adolescent psychiatry course utilising IACAPAP textbook and WHO mhGAP-IG in first year. clinical child and adolescent psychiatry internship in second year. both as part of master of science in integrated clinical and community mental health at Jimma University, Ethiopia	The Ethiopian program

### Program Descriptions

*The Brazilian program* (Blanco-Vieira et al., 2017)—The authors studied the CESMIA program, an 18 month (360 h) postgraduate course which used a problem-based learning method. CESMIA catered to professionals from multiple disciplines with the main goal of training those involved in the care of children and adolescents with mental health problems, preferably in the public system (Table 3 in Appendix).

*The Ugandan program* (Akol et al., 2017)—The authors evaluated a short in-service five day program for clinical officers, nurses and midwives conducted in a psychiatric facility in Uganda. The program provided training on the screening, assessment and management or referrals of children and adolescent with mental health problems using a curriculum based on the mhGAP-IG version 1.0 and PowerPoint slides from IACAPAP (Table 3 in Appendix).

*The Nigerian program* (Omigbodun et al., 2007)—The paper evaluated a short three day course in child and adolescent mental health conducted by The West African College of physicians (Nigerian Chapter), Faculty of Psychiatry. The course was aimed at a multidisciplinary audience to inform future training, and was attended by nurses, psychiatrists, family medicine specialists, paediatricians, psychologists and community health workers (Table 3 in Appendix).

*The Varanasi Indian program* (Dogra et al., 2005)—The authors of this study discussed and evaluated a child mental health program delivered in Varanasi, India, which was jointly developed by the University of Leicester, UK, Northampton Child and Family Consultation Service, UK, and Benares Hindu University, India. This seven day program was a modified version of the undergraduate child psychiatry training program for medical students in the University of Leicester. The training cohort included psychiatrists, junior medical officers, psychologists, nurses, social workers and student social workers (Table 3 in Appendix).

*The SAPP Indian program* (Russell & Nair, 2010a, 2010b)—Described over two articles, the authors evaluated the strengthening of the paediatricians project (SAPP) conducted under the national task force on family life and life skill education (NFLLSE) of the Indian academy of paediatrics. This three day program focused on up-skilling paediatricians in identifying and treating the priority mental health disorders (PMHD) among adolescents as part of a larger Postgraduate Diploma in Adolescent Paediatrics by Child Development Centre and University of Kerala. Trainees included specialist paediatricians, junior doctors with interest in paediatrics, as well family medicine practitioners (Table 3 in Appendix).

*The Ethiopian program* (Tesfaye et al., 2014)—The authors described the development and implementation of child psychiatry training within the master of science program in Integrated Clinical and Community Mental Health at Jimma University in Ethiopia. The program development was a collaborative effort between Addis Ababa University, Jimma University and Amanuel Mental Specialised Hospital, all in Ethiopia, along with Lugwig-Maximilians University in Munich, Germany. The child and adolescent mental health training occurred in two separate blocks (2 weeks and 4 weeks) over 2 years. The course catered to non-physician clinicians, particularly health officers and nurses with undergraduate degrees and trained to provide primary health care (Table 3 in Appendix).

### Educational Resources Used

Both the Ugandan and Ethiopian programs based their training on material from the WHO’s mhGAP-IG, as well as IACAPAP resources that had been contextualised to the local environment and needs (Blanco-Vieira et al., 2017; Tesfaye et al., 2014). The mhGAP-IG’s goals include the up-scaling of services for mental, neurological and substance use disorders especially for LMICs, and with a focus on PMHD (Keynejad et al., 2018). With this in mind, the mhGAP-IG was developed through an extensive international consultative process arriving at assessment and treatment algorithms adapted for local use by health care workers working in non-specialised settings (Keynejad et al., 2018). The freely available electronic IACAPAP textbook and resources are described as having been collaboratively developed with over 100 contributors internationally, and used extensively in LMICs (Coughlan & Perryman, 2015; Rey & Omigbodun, 2015).

Three of the courses, the Nigerian, the Brazilian and the SAPP Indian programs, were all described as entirely local initiatives (Blanco-Vieira et al., 2017; Omigbodun et al., 2007; Russell & Nair, 2010a, 2010b). However, additionally the SAPP Indian program referenced using the WHO’s descriptions of the PMHD of adolescence as the guide in course development (Russell & Nair, 2010a, 2010b). On the other hand, the Ethiopian and Varanasi Indian programs were both described as being developed through international collaborations with institutions in high income countries, but with local adaptations: the Ethiopian program was developed in collaboration with a German institution and the Varanasi Indian program in collaboration with British institutions (Dogra et al., 2005; Tesfaye et al., 2014).

Whilst it is not possible with the available information to definitively determine the most appropriate CAMH educational resource for a training program in each specific LMIC setting, it is conceivable to consider other important factors. In this way, the global accessibility, recognised quality, and low costs of utilising the mhGAP-IG along with IACAPAP resources would suggest that all training initiatives in LMICs will likely benefit with incorporating these with the appropriate local adaptations.

### Teaching Methods

All courses were described as using a mixture of didactic teaching along with interactive teaching styles, such as class discussions, supervised smaller group based case discussions, and role plays (Akol et al., 2017; Blanco-Vieira et al., 2017; Dogra et al., 2005; Omigbodun et al., 2007; Russell & Nair, 2010a, 2010b; Tesfaye et al., 2014). Two of the courses additionally allowed for learning through clinical exposure ranging from the Ugandan program’s 2 h of clinical demonstration in the children’s ward to the Ethiopian program’s training using video recordings of child psychiatric outpatient interviews and observations in the first year followed by a four week clinical internship in an outpatient clinic in the second year (Akol et al., 2017; Tesfaye et al., 2014). In relation to ethical concerns regarding videotapes of children attending the psychiatric outpatient clinic incorporated into the Ethiopian program, the authors reported that they obtained “parental consent and child assent” from participating patients and families prior to recording (Tesfaye et al., 2014).

Five studies provided information on participant perspectives of teaching methods. In the Ethiopian study and the Varanasi Indian study, participants reported feedback that interactive case based discussions and the use of role play was very useful (Dogra et al., 2005; Tesfaye et al., 2014). The Varanasi Indian study reported that participants would have appreciated more video and live case presentations of local cases along with a demonstration of assessment techniques using a patient (Dogra et al., 2005). The SAPP Indian study used video recordings of role play interviews for later analysis, and the authors reported that this was appreciated by participants (Russell & Nair, 2010a, 2010b). Participants in the Nigerian program felt that video recordings of children presenting with mental disorders would have been helpful (Omigbodun et al., 2007). The common theme in the feedback received from participants from different programs was for greater clinical exposure or demonstrations using “real life” cases. This appears to be important in meeting trainee satisfaction and the ethical challenges could be overcome by taking appropriate consent in the context of local cultural challenges.

### Course Scheduling

Some studies included participant feedback and course evaluations pertaining to time management (Dogra et al., 2005; Omigbodun et al., 2007; Tesfaye et al., 2014). In the 3 day long Nigerian program, the majority (76%) of participants felt that the covered topics required more time. Some participants suggested extending the course by 1–2 days and others suggested more frequent installations of the course to enable them to learn more on the subject (Omigbodun et al., 2007). At 7 days duration, the Varanasi Indian course participants highlighted difficulties with the timetable due to it being tight and inflexible, and considered that it would have been better if they had scheduled more time for reflection (Dogra et al., 2005). While in the Ethiopian course, which was part of a larger Master’s degree and was 2 weeks long in the first year followed by a 4 week clinical internship in the second year, trainee feedback again highlighted that the course duration was too short (Tesfaye et al., 2014). This study provided data on three cohorts with modifications to course teaching in each subsequent first-year program, and trainees’ rating of the duration improved after the first cohort. With the second cohort, increased time was scheduled to discuss patient management whilst classroom teaching time was decreased to allow for more clinical activities and in the third cohort, the program was modified and extended to 3 weeks to allow for further focus on problem based decision making and management (Tesfaye et al., 2014). The authors reported that compared to previous years, there was more time to do in-depth discussion of patient management (Tesfaye et al., 2014). In considering all of the above information, the prominent themes are that careful time management is important, and particular efforts should be made to ensure that adequate time is tabled to allow for reflection and case discussion.

### Course Content

Four of the studies presented information on course content feedback (Omigbodun et al., 2007; Tesfaye et al., 2014). Participant evaluation of the Ethiopian program was generally positive, however trainees suggested that a quick overview of the relevant diagnostic systems would be helpful at the end of case discussions (Tesfaye et al., 2014). In later follow up of trainees who had graduated from the program, they had suggested decreasing the overall course content and increasing focus on specific areas such as enuresis and epilepsy (Tesfaye et al., 2014). Most participants in the Nigerian program were pleased with the course content and reported that it met their expectations to learn the principles of assessment and management, along with skills in the early detection of CAMH illnesses within local contexts (Omigbodun et al., 2007). Trainees also wanted to increase their knowledge of physical and emotional development and be able to educate families on normal developmental processes in children and adolescents. Some participants highlighted the need for additional sessions on stigma and forensic mental health (Omigbodun et al., 2007). In the SAPP Indian program, the participants reported that education on psychopathology, mental status examination and the management of PMHD was important, however they wanted further focus on diagnostic systems (Russell & Nair, 2010a, 2010b). Participants also reported a desire for content addressing more commonly diagnosed issues in general paediatric practice such as stammering and breath-holding spells. Further to this, trainees also wanted to gain knowledge in neurodevelopmental disorders (Russell & Nair, 2010a, 2010b). The common theme from the participant feedback in these courses was that participants wished to have more content focused on clinical case discussions with real cases relevant to the local contexts. Additionally, participants wanted a focus on the application of diagnostic systems and management in case discussions. They also expressed a need to understand and address developmental concerns in children and adolescents (Omigbodun et al., 2007; Russell & Nair, 2010a, 2010b; Tesfaye et al., 2014).

### Trainee Evaluation Tools

Information on tools used for evaluation of course participants was included in six of the seven studies (Akol et al., 2017; Blanco-Vieira et al., 2017; Omigbodun et al., 2007; Russell & Nair, 2010a, 2010b; Tesfaye et al., 2014). Of these, five included pre- and post-testing methods (Akol et al., 2017; Blanco-Vieira et al., 2017; Omigbodun et al., 2007; Russell & Nair, 2010a, 2010b; Tesfaye et al., 2014). The Brazilian program incorporated a knowledge, attitudes and practice (KAP) pre-test and post-test questionnaire composed of 18 questions with responses recorded on a Likert scale format (Blanco-Vieira et al. 2017). Although student results significantly improved in all domains between pre- and post-tests, the authors highlight that this KAP questionnaire was a self-report tool and not a validated assessment instrument (Blanco-Vieira et al., 2017). In the Ugandan program, the test administered for pre- and post-testing was derived from a standardised CAMH assessment tool included in the mhGAP-IG for trainings (Akol et al., 2017). This was to study the changes in knowledge and attitudes and used a combination of binary true/false and multiple choice questions along specific themes covering the curriculum. The authors reported that the construct and face validity of the tool were determined on the basis of expert opinion and prior use in the sub-Saharan setting (Akol et al., 2017). Mean scores for all participants in the Ugandan program improved between both tests (Akol et al., 2017).The SAPP Indian study also tested participants on knowledge acquisition with a pre- and post-test assessment of the trainees (Russell & Nair, 2010a, 2010b). A brief test with the same five multiple choice questions was administered on both occasions covering course curriculum. Improvement was observed in all responses at post-testing reaching statistical significance in four out of the five questions. The authors did not comment on the validity of their assessment tool (Russell & Nair, 2010a, 2010b). In the Nigerian program, participants completed pre- and post-test knowledge based multiple choice questionnaires, which registered a significant improvement in mean scores (Omigbodun et al., 2007). No comments were provided regarding the validity of the assessment tools used.

The trainees in the first year of the Ethiopian program were assessed through observations of clinical work and seminar presentation skills (Tesfaye et al., 2014). Feedback was then provided to the trainees individually and in groups focusing on strengths and areas for further improvement. They also underwent two written tests and grades were provided based on these tests along with their class contributions. All trainees had results ranging from “good to excellent”. The trainees in the second year of the program underwent a supervised clinical internship and were assessed using an adapted version of a structured local clinical assessment tool used to assess first year medical residents. Similar to results obtained in the first year of the program, all trainees who had completed the internship passed with results ranging from “good to excellent” (Tesfaye et al., 2014).

It is difficult to determine the effectiveness of a program without long-term clinical practice outcome data. However, among the programs studied here, the Ethiopian program’s use of clinical and teaching skill assessments during the course is the most thorough in examining the practice of trainees. Obtaining pre- and post-test data on knowledge gained is important for performing course analyses. Where possible, it will be important to use a validated tool. As the mhGAP-IG already includes such a tool, this could be adapted to the local context following the Ugandan program’s experience.

## Discussion

This study critically reviewed seven articles which discussed six different programs that conducted general CAMH training for non-specialist health professionals in LMICs.

In relation to the educational resources used, the included programs incorporated both local and international resources. Due to insufficient detail on source materials, a comparative analysis of all the resources used was not possible. However, two of these programs used the WHO’s mhGAP-IG along with online IACAPAP resources, adapted to suit the local contexts (Blanco-Vieira et al., 2017; Tesfaye et al., 2014). The mhGAP-IG has been described as evidence based, tailored especially to the identification and management of PMHDs as designated by the WHO and specifically targeting a non-specialised audience, thus catering to primary health care in LMICs (Keynejad et al., 2018). The IACAPAP online textbook and associated online resources have been lauded for the extensive international collaboration and contribution, as well as for its focus on self-directed learning (Coughlan & Perryman, 2015; Rey & Omigbodun, 2015). Both of these can be described as open educational resources (OER), and this form of knowledge sharing is considered to be highly beneficial in the training of professionals in resource poor countries (Coughlan & Perryman, 2015). Another reason to consider freely available electronic resources is the growing access to online media via the rapidly expanding usage of smartphones in LMICs. This would permit access to powerful resources at the point of care despite geographical and financial limitations (Rey & Omigbodun, 2015). This is further supported by the growing availability of smartphones in multiple languages, increasing accessibility to more LMIC professionals (Keynejad et al., 2018; Rey & Omigbodun, 2015). It is important to note that whilst these resources were used in two of the programs, they required modifications to suit the local context suggesting they may not be able to completely satisfy all settings and needs in the developing world. This informs the recommendation that whilst a variety of source materials may be consulted upon in the development of a CAMH training program in a LMIC, it will be important to include adaptations of the mhGAP-IG and IACAPAP resources given their extensive benefits.

This study identified perspectives on educational strategies. Whilst both didactic and interactive teaching methods were valued, participants were most appreciative of clinical case exposure through case based discussions, role plays and clinical demonstrations. Where this was not included adequately, there appeared to be a common and prominent feedback requesting further inclusions in future courses. This is consistent with the recommendation that with the training of non-specialist health workers in CAMH, it is important to ensure that the training is relevant to their local context (Patel et al., 2008). Additionally, it will be important for course organisers to consider adult learning principles in their teaching methods including experiential learning, the need for adult students to understand and know why they are learning the material, as well as to be actively involved in the learning process given that adults are motivated by their need to problem solve (Bryan et al. 2009). All of these are consistent with the overall need and preference of trainees for greater clinical exposure and case and problem-based learning. Future CAMH training courses should strongly consider how they will be able to cater effectively for this whilst addressing the ethical concerns relating to the involvement of vulnerable young people and their families. Furthermore, it may be beneficial for future courses to adopt a ‘train the trainer’ component to support continual learning, and/or to consider how longer component could be incorporated that allows feedback and advanced learning as trainees use the knowledge obtained during the program and gain on the job experience.

When looking at course durations, the different programs included in this study comprised a large range of time frames owing to their different curriculums and purposes. This made comparative discussion challenging. However, through analysis of participant feedback, the prominent theme of a need for less intense and more flexible timetables to allow space for reflection emerged. Active learning as opposed to passive learning, emphasises interactive learning with a focus on both action and reflection, and this has been reported to be useful for acquiring skills that will be necessary within the context of clinical work (Beidas & Kendall, 2010). With this in mind, it will be important for the developers of future CAMH training courses in LMICs to allow for additional time for discussion of cases, answering of questions, and reflection, rather than to only prioritise the insertion of academic material in the schedule.

In the exploration of trainee requests for course content adaptations, themes that emerged were the need to understand developmental concerns and neurodevelopmental disorders, as well as focus on the local contexts, and the application of diagnostic systems and management plans. This may suggest the overall need and desire of trainees to understand and learn the practical application of core CAMH skills as it applies to their local contexts. It has been recommended that training organisers should ensure that the curriculum is tailored to meet the competencies required locally for “effective and successful practice” (Hoge et al. 2004). Tailoring courses to address these concerns could heighten trainee confidence in their ability to transfer their knowledge into their clinical work.

Several types of pre- and post-test assessments were used in trainee assessments. Whilst there was consistent improvement in results between tests, the validity was not commented upon in the majority of programs evaluated in this study. The only exception to this was the Ugandan program where authors reported that they had adapted an assessment tool included in the mhGAP-IG to the local context, and described addressing the face and construct validity of this tool through its use in the sub-Saharan context and on expert appraisal (Akol et al. 2017). The use of a validated instrument such as the mhGAP-IG tool will be important in gauging the effectiveness of knowledge gained but it will also allow for comparison between courses, especially if a number of different programs utilise the same tool. This positively addresses the issue of an effective iterative process in program development over time. Programs can learn more from each other if assessment tools are standardised across programs and consider how the differences in the programs may have contributed to the different results (Beidas & Kendall, 2010). It would appear that standardising assessment tools would assist with this and to this end the mhGAP-IG instrument appears most suited for this purpose at least from the studied programs.

As part of this iterative process, it will also be important for continual collection of trainee feedback. It has been recommended that regular reviews of the curriculum occur to ensure that they meet the needs of the trainees (Hoge et al. 2004). Whilst educational organisations and accreditation agencies should conduct their own reviews, processes should try to accommodate trainee views potentially 1–2 years after course completion to allow for sufficient use of the knowledge and skills gained in their clinical work (Hoge et al. 2004).

There are several limitations of the study. Firstly, the exclusion of non-English language publications would potentially lead to the exclusion of peer-reviewed studies from several of the non-English speaking LMICs. Secondly, the databases used in the search may not have included all relevant studies. Thirdly, the exclusion of non-peer reviewed papers or reports may have led to the exclusion of information from a number of programs in developing countries. The International Child and Adolescent Mental Health (ICAMH) initiative of IACAPAP has organised a number of training programs in LMICs. Unfortunately, descriptions of their efforts in IACAPAP online media do not have sufficient useful detail to permit the scrutiny required for this study. Fourthly, a comparison was not done with training efforts in non-LMICs, which may have revealed further information on best practices in developing CAMH training courses. Lastly, whilst a scoping study does provide a narrative account of current practices, it is difficult to quantitatively consider the weight of evidence as compared to systematic reviews (Arksey & O’Malley, 2005). Search methods yielded just seven studies discussing information from only six programs across three continents and these programs were vastly different to each other in terms of their source materials, durations, contents, teaching methods and evaluation styles, making it very challenging to accurately compare and evaluate them and apply recommendations across all the LMICs with similar outcome expectations.

There are several strengths of the study. Firstly, the use of a scoping study allowed for qualitative review of data, and this was relevant especially as a large aspect was the appraisal of subjective participant feedback. Secondly, the inclusion of only peer reviewed data allowed for comparison between programs with a higher quality of information and this allowed for greater analysis. Thirdly, a published review of several CAMH training initiatives is currently unavailable and this may be the first time a review of such programs in LMICs has taken place and despite the challenges, useful information has been obtained to inform future efforts in the area. If the suggestions are followed especially with regard to standardising trainee assessments across programs, this will also encourage rapid quality improvement as courses across different LMICs can learn from each other.

## Conclusions

In conclusion, a number of recommendations (Table 2) have arisen from this study for future CAMH training programs. These include the need to incorporate mhGAP-IG and IACAPAP educational resources, the importance of allowing additional time for discussion and reflection, and focusing more on the learning of the practical applications of core CAMH skills through local case based learning with ethical considerations. Another recommendation is to ensure the inclusion of pre and post-test trainee evaluations ideally using a validated tool that has greater potential for wider acceptance among LMICs such as the mhGAP-IG tool. It is hoped that the organisers of future CAMH training in developing countries can institute some of the recommendations in future courses. Further studies may then assist in addressing this huge challenge. This will be important in shaping the work force needed to address the mental health treatment gap for some of the most vulnerable groups of people in the world.

**Table 2:** Study recommendations

Area	Recommendations
Educational resources	Include mhGAP-IG and IACAPAP resources in source materials
Teaching methods	Include more case based learning and problem solving based on local cases with consideration for ethical concerns
Course scheduling	Ensure adequate time is allocated for case discussion and reflection
Course content	Include a focus on practical understanding and application of core CAMHS skills in diagnosis, management and developmental concerns to suit local contexts
Trainee evaluation	Do pre- and post-test assessments. Utilise a validated trainee assessment tool that is likely to be more widely used allowing for greater comparative analyses and opportunities for learning. At this stage, the most appropriate tool appears to be that which is included in the mhGAP-IG. mhGAP-IG training material and the assessment tool is available through the following link: https://www.who.int/mental_health/mhgap/trainingmanuals_tohp_cmh/en/

## Appendix

See Table

**Table 3:** Program descriptions

Program	Course content
The Brazilian program (Blanco-Vieira et al., 2017) CESMIA	Foundations of children and adolescents’ mental healthcare Normal development Child and adolescent psychopathology Interventions for child and adolescent mental health disorders Substance use disorders and vulnerability to environmental risk factors Management of public health services for children and adolescents Seminars about research methodology
The Ugandan program (Akol et al., 2017)	Introduction to child and adolescent mental health and mhGAP-IG Overview of aetiology, diagnosis and treatment planning for child and adolescent mental health problems, symptomatology and terminology Normal development in infancy, childhood and adolescence Attachment and positive parenting considering parental mental illness The clinical examination of children, adolescents and their families Psychosocial treatments Paediatric psychopharmacology School underachievement, specific learning difficulties and intellectual disability Externalising disorders [attention deficit hyperactivity disorder (ADHD), conduct disorder and oppositional defiant disorder] and managing difficult behaviour Autism spectrum disorders Enuresis and encopresis Epilepsy Depression, suicide and self-harming behaviour Bipolar disorder and Psychosis Tic disorders Anxiety disorders, separation anxiety, obsessive compulsive disorder (OCD), post traumatic stress disorder (PTSD), somatoform disorders Bereavement Physical illness and mental health (HIV/AIDS, diabetes, sexually communicable diseases, etc.) Alcohol use disorders, substance use disorders and internet addiction
The Nigerian program (Omigbodun et al., 2007)	Public heath aspects of child mental health Normal development (infancy, childhood and adolescence) Aetiological influences on child psychiatric conditions (genetic, environmental) Psychiatric assessment of the infant, child and adolescent Psycho educational assessment of children and adolescents (learning disorders, learning disability) Child psychiatry and paediatrics (collaboration and consultation-liaison) Child psychiatry in primary care and general practice (collaboration, consultation-liaison, prevention and early detection) Common syndromes in child and adolescent psychiatry Overview of child and adolescent neurology (evaluation and diagnosis, epilepsy) Management issues in child psychiatry Psychotherapy (child, parent–child relationship, family therapy) Child psychopharmacology Care of orphans and vulnerable children—psychosocial issues
The Varanasi Indian program (Dogra et al., 2005)	Defining mental health (considering stigma) Descriptions of CAMHS services in the UK and referral processes Child development Family development and life events that impact on the mental health of young people Assessing child mental health and special considerations for learning disabilities Autism and ADHD Anxiety disorders (including PTSD and adjustment disorders) Eating disorders and depression Psychosis and major mental illness Obsessive compulsive disorder Treatment strategies in the primary care context (parenting interventions, behavioural management, relaxation, supportive care, and reframing) Discussions on models for training and improving access to and developing child mental health services
The SAPP Indian program (Russell & Nair, 2010a, 2010b)	Phenomenology Psychopathology using case vignettes and role-play Introduction to international statistical classification of diseases and related health problems 10th revision (ICD-10) Making ICD-10 diagnoses using the psychopathology identified in the role-plays Pharmacotherapy for priority mental health disorders (PMHD) Psychotherapy for PMHD
The Ethiopian program (Tesfaye et al., 2014)	Pervasive developmental disorders Intellectual disability Childhood behavioural and emotional disorders Enuresis ADHD Child abuse and neglect Adolescent medicine issues such as sexual development, drug use, and risk factors and prevention of suicide

3.
